# The Ileal Lipid Binding Protein Is Required for Efficient Absorption and Transport of Bile Acids in the Distal Portion of the Murine Small Intestine

**DOI:** 10.1371/journal.pone.0050810

**Published:** 2012-12-10

**Authors:** Dana Praslickova, Enrique C. Torchia, Michael G. Sugiyama, Elijah J. Magrane, Brittnee L. Zwicker, Lev Kolodzieyski, Luis B. Agellon

**Affiliations:** 1 School of Dietetics and Human Nutrition, McGill University, Ste. Anne de Bellevue, Québec, Canada; 2 Canadian Institutes of Health Research Group in Molecular and Cell Biology of Lipids, University of Alberta, Edmonton, Alberta, Canada; 3 ITR Laboratories Canada, Baie D'Urfe, Québec, Canada; University of Bari & Consorzio Mario Negri Sud, Italy

## Abstract

The ileal lipid binding protein (ilbp) is a cytoplasmic protein that binds bile acids with high affinity. However evidence demonstrating the role of this protein in bile acid transport and homeostasis is missing. We created a mouse strain lacking ilbp (*Fabp6^−/−^* mice) and assessed the impact of ilbp deficiency on bile acid homeostasis and transport in vivo. Elimination of ilbp increased fecal bile acid excretion (54.2%, *P*<0.05) in female but not male *Fabp6^−/−^* mice. The activity of cholesterol 7α-hydroxylase (cyp7a1), the rate-controlling enzyme of the classical bile acid biosynthetic pathway, was significantly increased in female (63.5%, *P*<0.05) but not in male *Fabp6^−/−^* mice. The amount of [^3^H]taurocholic acid (TCA) excreted by 24 h after oral administration was 102% (*P*<0.025) higher for female *Fabp6^−/−^* mice whereas it was 57.3% (*P*<0.01) lower for male *Fabp6^−/−^* mice, compared to wild-type mice. The retained fraction of the [^3^H]TCA localized in the small and large intestines was increased by 22% (*P*<0.02) and decreased by 62.7% (*P*<0.01), respectively, in male *Fabp6^−/−^* mice relative wild-type mice, whereas no changes were seen in female *Fabp6^−/−^* mice. Mucosal to serosal bile acid transport using everted distal gut sacs was decreased by 74% (*P*<0.03) in both sexes of *Fabp6^−/−^* mice as compared to wild-type mice. The results demonstrate that ilbp is involved in the apical to basolateral transport of bile acids in ileal enterocytes, and is vital for the maintenance of bile acid homeostasis in the enterohepatic circulation (EHC) in mice.

## Introduction

Bile acids are biological detergents produced by the liver that are needed for the absorption of dietary lipids and lipid-soluble nutrients from the lumen of the small intestine. These amphipathic molecules are efficiently recovered by ileal enterocytes and returned to the liver via the portal vein. Bile acids typically go through several cycles of reuse within the EHC, and undergo structural modifications before being excreted from the body. Targeted deletion of the murine *Slc10a2* gene, which encodes the ileal apical sodium-dependent bile acid transporter (asbt), and the phenotype of non-functional variants of the human *SLC10A2* gene have established that asbt is the primary membrane-bound transporter involved in the active re-uptake of bile acids in the small intestine [1], [2]. Efflux of bile acids from enterocytes to portal blood is now known to be mediated by a heteromeric membrane-bound transporter composed of organic solute transporter (ost) α and ostβ [3]. In mice, the deletion of the gene encoding ostα drastically decreases basolateral bile acid export, and increases fecal bile acid excretion [4], [5]. In contrast to the cellular import and export of bile acids, the mechanism for the transport of bile acids from the apical to basolateral membranes in ileal enterocytes is not clear.

The small intestine contains three intracellular lipid binding proteins: the liver fatty acid binding protein (L-FABP; gene symbol *Fabp1*), the intestinal fatty acid binding protein (I-FABP; gene symbol *Fabp2*), and the ileal lipid binding protein (ilbp; gene symbol *Fabp6*) [6]. L-FABP and I-FABP have long been thought to be involved in fatty acid metabolism. Ilbp can bind both fatty acids and bile acids, but exhibits greater affinity for bile acids [7], [8], [9], [10], [11]. Ilbp is found mainly in the distal portion of the small intestine, and its tissue localization coincides with that of both asbt and ostα/ostβ [3], [12]. These three proteins likely form the core components of the reclamation machinery for bile acids by the ileum, with ilbp involved in intracellular shuttling of bile acids. Using targeted gene ablation, we provide evidence showing that ilbp is required for efficient apical to basolateral transport of conjugated bile acids in murine ileal enterocytes.

## Results

### Disruption of the Murine *Fabp6* Gene

**Figure 1:**
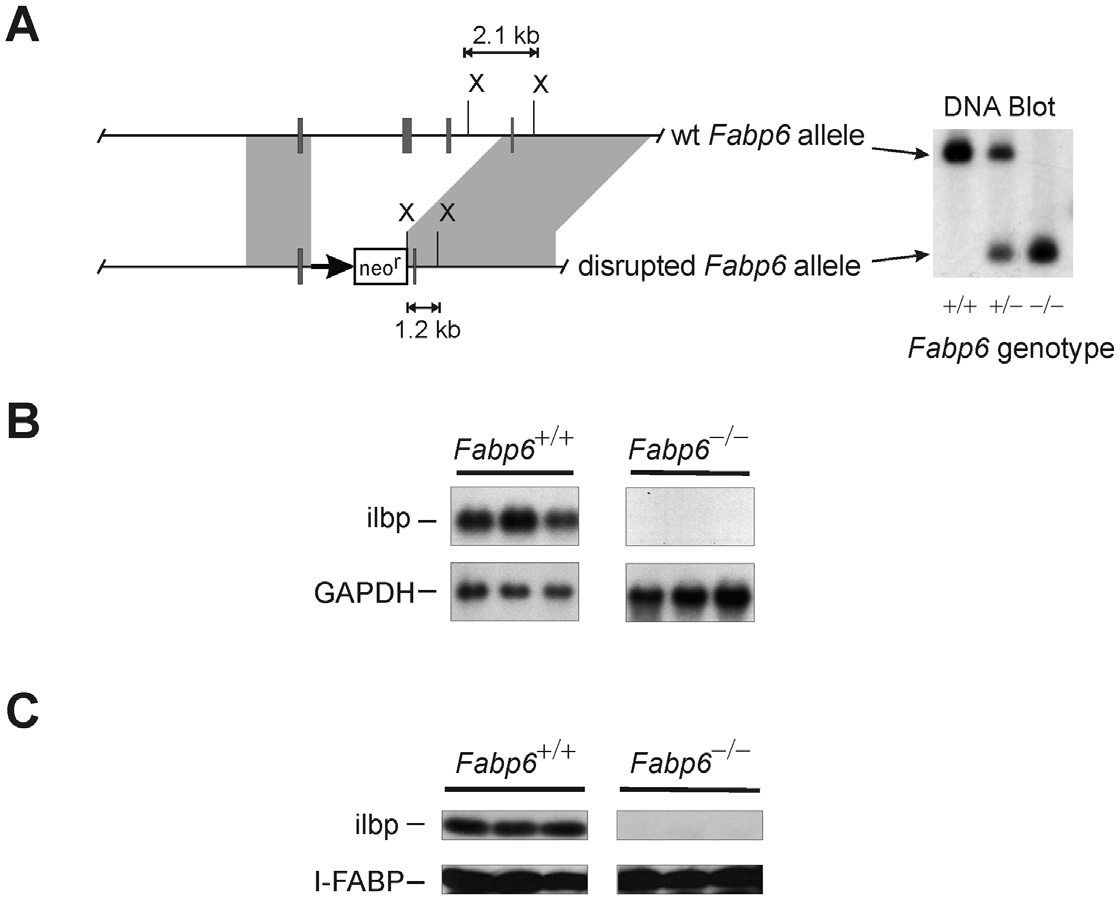
Targeted disruption of the murine *Fabp6* gene. (A) Structure of wild-type (wt) and disrupted *Fabp6* alleles. The targeting vector was designed to replace the region of the *Fabp6* gene encompassing exons 2 and 3 with the neo resistance (neo^r^) gene cassette. X, Xba I site. (B) RNA blot of small intestine RNA from *Fabp6* ^+/+^ and *Fabp6^−/−^* mice probed with [^32^P]-labeled ilbp cDNA. (C) Protein blot of small intestine homogenates from *Fabp6* ^+/+^ and *Fabp6^−/−^* mice probed with antiserum to murine ilbp.

Figure 1A shows the structure of the targeting vector used to inactivate the murine *Fabp6* gene. The targeting vector was designed to replace the region of the normal allele encompassing introns II to IV with the neomycin resistance gene cassette. The genotype of mice born from chimeric mice was confirmed by DNA blotting (Fig. 1A). Loss of the ilbp mRNA (Fig. 1B) and protein (Fig. 1C) in *Fabp6^−/−^* mice confirmed the inactivation of the *Fabp6* gene.

### General Features of Ilbp-deficient Mice

*Fabp6^−/−^* mice were viable and showed no overt signs of abnormalities. Body weights of 17–20 week old wild-type (*Fabp6^+/+^*)and *Fabp6^−/−^* mice were comparable (male *Fabp6^+/+^* mice, 26.1±1.7 g vs. male *Fabp6^−/−^* mice, 25.4±1.6 g; female *Fabp6^+/+^* mice 21.9±2.8 g vs. female *Fabp6^−/−^* mice, 20.5±1.2 g; n = 5 per group). Intestinal length was also comparable (male *Fabp6^+/+^* mice, 37.2±1.5 cm vs. male *Fabp6^−/−^* mice, 36.4±0.7 cm; female *Fabp6^+/+^* mice, 35.4±1.5 cm vs. female *Fabp6^−/−^* mice, 35.0±1.0 cm; n = 5 per group). The liver weight to body weight ratio tended to be lower in male ilbp-deficient mice (male *Fabp6^+/+^* mice, 0.047±0.001 vs. male *Fabp6^−/−^* mice, 0.044±0.002; n = 5 per group) and was significantly reduced in female ilbp-deficient mice (female *Fabp6^−/−^* mice, 0.045±0.001 vs. female *Fabp6^−/−^* mice, 0.042±0.001, P<0.05; n = 5 per group).

Analysis of plasma lipids of mice fed the standard lab diet showed a decrease in both total cholesterol and triacylglycerol concentration in male, but not in female *Fabp6^−/−^* mice (Table 1). The lipid profile determined by separation of plasma lipoproteins by HPLC showed that the loss of ilbp did not cause significant changes in distribution of lipids in lipoprotein fractions (Fig. S1). Loss of ilbp had no effect on plasma glucose concentration or on plasma activities of both ALT and AST. Plasma bile acid concentration was comparable in male wild-type and *Fabp6^−/−^* mice, but was increased in female *Fabp6^−/−^* mice relative to wild-type *Fabp6^−/−^* mice. Histological analysis of ileal sections did not reveal remarkable changes in tissue morphology (Fig. S2). Similarly, no substantial difference was evident between liver of wild-type and *Fabp6^−/−^* mice (Fig. S3).

**Table 1:** Plasma chemistry.

Parameter	Males *Fabp6* ^+/+^	*Fabp6^−/−^*	Females *Fabp6* ^+/+^	*Fabp6^−/−^*
Total cholesterol (mg/dL)	63±13	48±6*	49±12	43±8
Total triacylglycerols (mg/dL)	46±11	34±10**	34±8	32±7
Glucose (mmol/L)	6.6±1.0	6.5±0.8	6.3±0.8	5.9±1.0
ALT (IU/L)	16.8±14.2	21.0±9.8	14.2±5.5	18.1±4.4
AST (IU/L)	37.5±15.2	70.5±69.3	68.5±50.0	64.1±44.9
Bile acids (µmol/L)	26.6±7.0	17.5±14.6	29.3±5.0	39.3±9.4*

* P<0.01,

** P<0.05, vs. wild-type, n = 10 per group. Values for ALT and AST are mean ± SEM.

### Bile Acid Metabolism in the EHC

To determine if loss of ilbp perturbed bile acid homeostasis, we analyzed indicators of bile acid metabolism in the EHC. The bile samples collected from all *Fabp6^−/−^* mice were clear and microscopic examination under polarized light did not reveal the presence of any birefringent substances. TCA and tauromuricholic acid accounted for >95% of bile acids in gallbladder bile. The TCA to tauromuricholic acid ratio was not affected by the loss of ilbp in both sexes (male *Fabp6^+/+^* mice, 1.86±0.86 vs. male *Fabp6^−/−^* mice, 2.40±1.22; female *Fabp6^+/+^* mice 2.64±0.78 vs. female *Fabp6^−/−^* mice, 2.03±0.10; n = 5 per group). The bile acid pool size was determined by measuring the total amount of bile acids in liver, gallbladder and small intestine. The loss of ilbp tended to decrease the bile acid pool size in the EHC of both male and female mice (Table 2). However fecal bile acid excretion rate increased by 55% in female *Fabp6^−/−^* mice but not in male *Fabp6^−/−^* mice (Table 2). These results indicate that female mice have a greater dependence on ilbp than male mice for the conservation of bile acids in the EHC.

**Table 2:** Bile acid pool size and excretion rate.

Parameter	Males *Fabp6* ^+/+^	*Fabp6^−/−^*	Females *Fabp6* ^+/+^	*Fabp6^−/−^*
Bile acid pool size (µmol/100 g body weight)	29.0±6.5	22.7±1.67	48.7±7.6	41.1±3.3
Fecal bile acid excretion (µmol/d/100 g body weight)	2.6±0.4	3.4±1.0	5.9±0.7	9.1±.9*

* P<0.02, vs. wild-type, n = 6 per group.

### Absorption of exogenously administered bile acid

**Figure 2:**
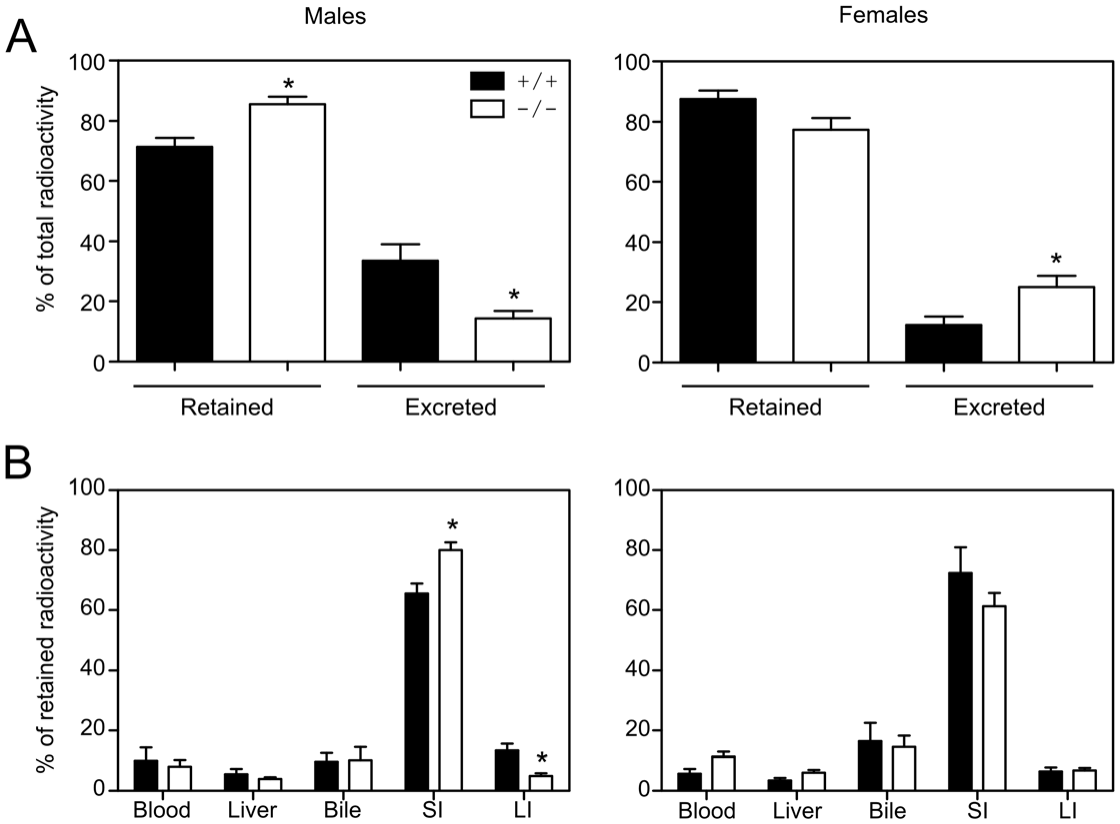
Absorption and tissue distribution of orally administered [^3^H]TCA in *Fabp6* ^+/+^ and *Fabp6^−/−^* mice. (A) Retained and excreted [^3^H]TCA at 24 h after oral administration. Values shown are mean±SEM (males, n = 4–5; females, n = 5–6). (B) Tissue distribution of the retained [^3^H]TCA in *Fabp6* ^+/+^ and *Fabp6^−/−^* mice. *P<0.05, **P<0.02, ***P<0.005, vs. wild-type of the same sex.

To visualize the movement of bile acids within the animal, [^3^H]TCA was administered by oral gavage and the distribution of the radioactivity was examined after 24 h. To minimize the dilution of the tracer by the endogenous bile acid pool, the total amount of administered TCA (25 µmol) was greater than the total amount of bile acids typically found in the EHC of mice as determined in this study (range = 3.9–17.6 µmol; median = 7.6 µmol; n = 32). The proportion of [^3^H]TCA excreted by male mice after 24 h was lower in male *Fabp6^−/−^* mice (by 57.3%, *P*<0.01; Fig. 2A), whereas it was higher (by 102%, *P*<0.025; Fig. 2A) in female *Fabp6^−/−^* mice, when compared to *Fabp6^+/+^* mice of the same sex. To determine the localization of the exogenously administered bile acid after 24 h, the distribution of [^3^H]TCA radioactivity was surveyed in vivo. [^3^H]TCA radioactivity was detected in the blood, liver and gallbladder (Fig. 2B and Fig. S4), as well as in urine (<2%). The percentage of the retained [^3^H]TCA radioactivity that was localized in the small intestine of male *Fabp6^−/−^* mice was 22% more (*P*<0.02; Fig. 2B) than in male *Fabp6^+/+^* mice, whereas the percentage of the retained [^3^H]TCA radioactivity localized in the large intestine was 62.7% less (*P*<0.01; Fig. 2B) in male *Fabp6^−/−^* mice. The loss of ilbp had no significant influence on the amount of [^3^H]TCA localized in the small or large intestine of female mice. These results indicate that the loss of ilbp impedes the movement of bile acids out of the small intestine of male mice.

The findings of the tracer study suggested that the loss of ilbp might affect the amount of tissue associated bile acids in small intestine. In age matched mice (12 weeks old), no difference between *Fabp6* ^+/+^ and *Fabp6^−/−^* mice of both sexes was evident in the wet weights of flushed small intestines (Fig. S5). The amount of tissue associated bile acids differed along the longitudinal axis with greater amounts in the distal third of the small intestine for both sexes of *Fabp6* ^+/+^ and *Fabp6* ^−/−^ mice. The loss of ilbp tended to decrease the amount of tissue associated bile acids in the proximal and medial segments in both sexes (Fig. S5).

### Bile acid transport across enterocytes

**Figure 3:**
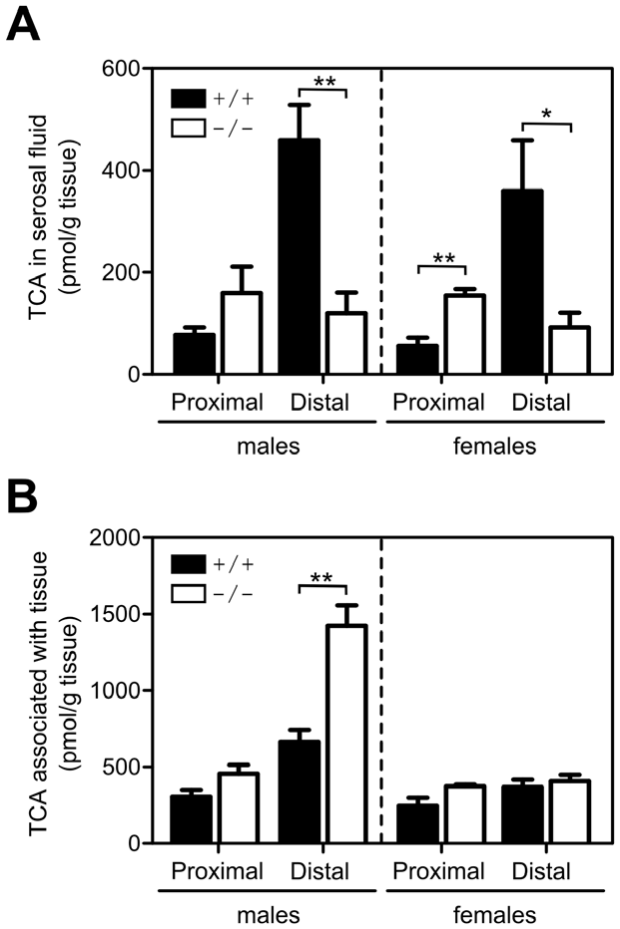
Transport of bile acids in everted gut sacs. (A) Amount of TCA accumulated in the serosal fluid after a 30 min incubation of gut sacs from the proximal one-third and distal one-third portions of the small intestine of male *Fabp6* ^+/+^ (n = 4), female *Fabp6* ^+/+^ (n = 3–4) mice (black bars), male *Fabp6^−/−^* (n = 4) and female *Fabp6^−/−^* (n = 5) mice (white bars). (B) Amount of TCA accumulated in the gut sac tissue at the end of the assay incubation period. *P<0.025, **P<0.01, vs. wild-type of the same sex.

To determine the transport of bile acids across enterocytes, the accumulation of TCA in serosal fluid in everted gut sacs was measured. Since both ilbp and asbt are known to be localized in the distal part of the small intestine, we used the corresponding proximal one-third portion of the small intestine to indicate bile acid transport activity in the absence of these proteins. As expected, the serosal fluid from the distal one-third portion of the everted small intestine from both male and female wild-type mice showed greater accumulation of TCA relative to the serosal fluid from the proximal one-third of the organ (Fig. 3A). The loss of ilbp drastically decreased the amount of radiolabeled bile acids recovered in serosal fluid by 73.9% (*P*<0.01; Fig. 3B) in males, and 74.3% (*P*<0.03) in females, as compared to wild-type mice. The amount of tissue-associated TCA was increased by 115.9% (*P*<0.01; Fig. 3B) in distal gut sacs of male *Fabp6^−/−^* mice compared to male wild-type mice, whereas no change was seen for those of female *Fabp6^−/−^* mice. These results illustrate that ilbp is essential for efficient transport of bile acids in ileal enterocytes.

### Indicators of Bile Acid Synthesis in the Liver

**Figure 4:**
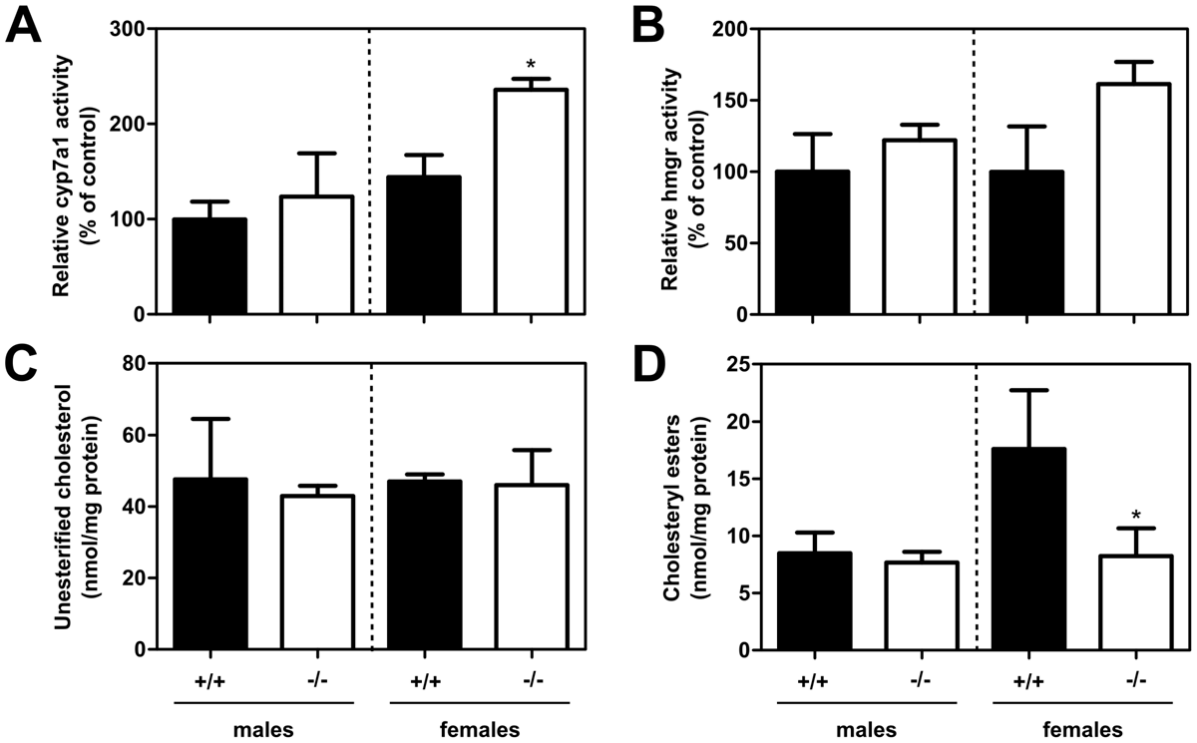
Hepatic cholesterol concentration and enzyme activities of *Fabp6* ^+/+^ (black bars) and *Fabp6^−/−^* (white bars) mice. (A) Unesterified cholesterol concentration and (B) Cholesteryl ester concentration, (n = 3). (C) Relative cyp7a1 activity (n = 5). (D) Relative HMGR activity (n = 5). Groups are compared relative to male *Fabp6* ^+/+^ mice.

**Figure 5:**
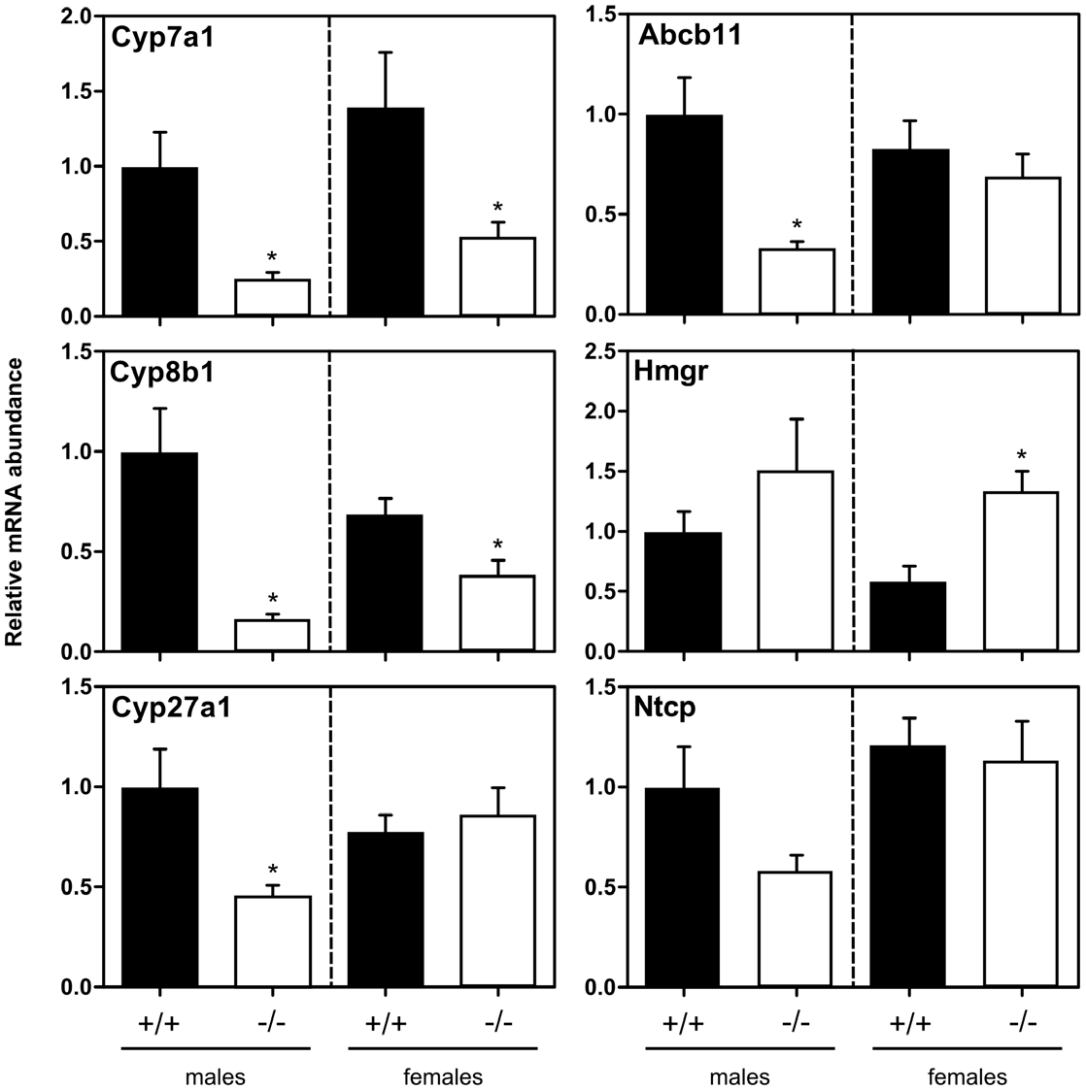
Survey of gene expression in the liver. qPCR analysis of liver RNA from *Fabp6* ^+/+^ (black bars) and *Fabp6^−/−^* (white bars) mice were done in duplicates. The abundance (mean ± SEM) of target mRNAs was normalized to internal standards and expressed relative male wild-type mice. *P<0.05, vs. wild-type.

Since bile acids can only be synthesized by the liver, the increased fecal bile acid excretion rate displayed by female *Fabp6^−/−^* mice predicted their increased rate of hepatic bile acid synthesis. In accordance, hepatic cyp7a1 enzyme activity in female *Fabp6^−/−^* mice was increased as compared to female *Fabp6* ^+/+^ mice (Fig. 4A). Normally, cyp7a1 mRNA abundance is a close predictor of cyp7a1 enzyme activity therefore the abundance of mRNA for cyp7a1 was measured by qPCR analysis. Contrary to expectation the cyp7a1 mRNA abundance was decreased in both sexes of *Fabp6^−/−^* mice, and this was accompanied by the decrease in cyp8b1 mRNA abundance (Fig. 5). It is known that *Cyp7a1* gene expression can also be inhibited in response to the fibroblast growth factor 15 (FGF15, gene symbol *Fgf15*) produced in the intestine. FGF15 mRNA abundance was increased in female *Fabp6^−/−^* mice but not male *Fabp6^−/−^* mice.

Next, hepatic unesterified and esterified cholesterol concentrations and hydroxymethylglutaryl-CoA reductase (HMGR) enzyme activity (Fig. 4B) were measured since bile acids are synthesized from cholesterol. Unesterified cholesterol concentration was comparable for both genotypes regardless of sex (Fig. 4C) whereas cholesterol ester concentration was decreased in female *Fabp6^−/−^* mice compared to female wild-type mice (Fig. 4D). The abundance of HMGR mRNA was also increased in female *Fabp6^−/−^* mice although the increase in HMGR activity measured in microsomes did not reach statistical significance. Taken together, the results support the concept of increased demand for cholesterol in female *Fabp6^−/−^* mice that would be expected due to increased bile acid synthesis.

### Expression of Bile Acid Transporter Genes within the EHC

**Figure 6:**
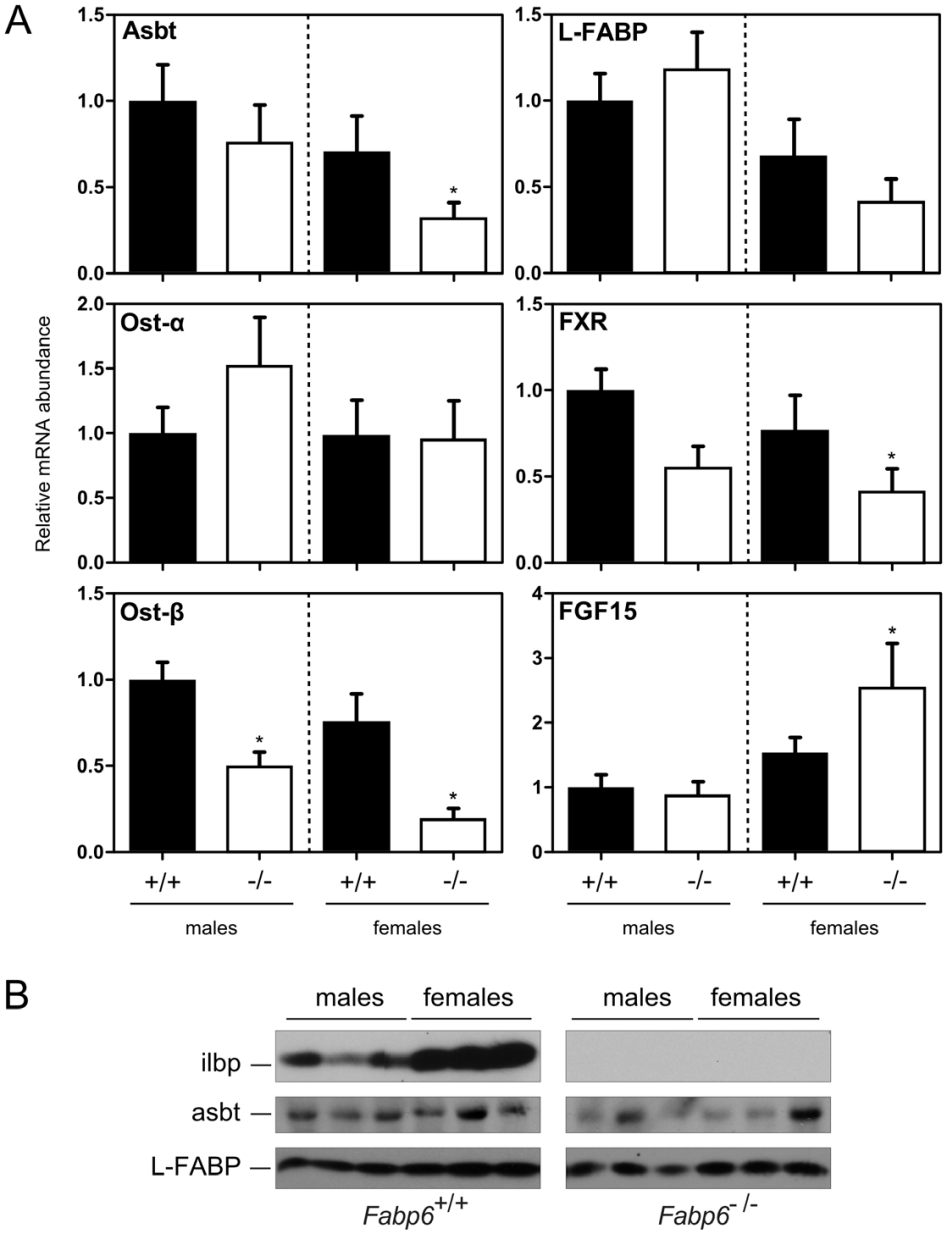
Survey of gene expression in the small intestine. (A) qPCR analysis of RNA from small intestines of *Fabp6* ^+/+^ (black bars) and *Fabp6^−/−^* (white bars) were done in duplicates. The normalized abundance (mean±SEM) of target mRNAs is expressed relative to male *Fabp6* ^+/+^ mice. *P*<0.05, vs. wild-type of the same sex. (B) Protein blots of small intestine homogenates of *Fabp6* ^+/+^ and *Fabp6^−/−^* mice were probed with antisera to ilbp, asbt and L-FABP.

Analysis of mRNA abundance for major hepatic and intestinal bile acid membrane-bound transporters did not reveal an overall pattern of alteration in response to ilbp deficiency that was consistent to both males and females. In the liver, sodium/taurocholate cotransporting polypeptide (ntcp; gene symbol *Slc10a1*) and abcb11 mRNA were decreased but only in males (Fig. 5). In the intestine, the loss of ilbp caused variable asbt mRNA abundance (Fig. 6A), however asbt protein abundance measured in homogenates of whole small intestine was decreased. In contrast, no change in L-FABP protein abundance was observed (Fig. 4B). No change in ostα mRNA abundance was found but ostβ mRNA was decreased in both male and female *Fabp6^−/−^* mice (Fig. 6B). The proximal to distal distribution of asbt mRNAs was not altered by the loss of ilbp, whereas the distribution of ostα mRNA expand towards the medial portion of the small intestine but only in males (Fig. S6). Interestingly, ilbp is present in greater amounts in the small intestine of female wild-type mice compared to male wild-type mice (Fig. 6B).

## Discussion

The identity of major transporters involved in the movement of bile acids within the EHC has been determined by targeted gene disruption in mice [2], [4], [13]. Removal of asbt causes a defect in the import of bile acids by ileal enterocytes resulting in severe bile acid malabsorption shrinking the bile acid pool size which forces the liver to increase bile acid synthesis to compensate for the loss [2]. Since bile acids no longer re-enter the body, *Cyp7a1* gene expression is derepressed as bile acid-mediated negative feedback inhibition of *Cyp7a1* gene expression, involving the small heterodimer partner (SHP, gene symbol *Nr0b2*) in hepatocytes, or involving FGF15 from the ileal enterocytes, does not occur. Males and females have similar patterns of change but severity differs between the two sexes. Similarly, targeted deletion of the murine gene for ostα disrupts bile acid homeostasis in the EHC [4], [5]. This finding parallels previous studies showing that both ostα and ostβ are required to form a functional basolateral bile acid transporter [3]. The bile acid pool size in mice lacking ostα is dramatically reduced, but the rate of fecal bile acid excretion is unaffected due to the inhibition of bile acid synthesis through fibroblast growth factor 15-mediated repression of *Cyp7a1* gene expression. As in the case of asbt deficiency, the pattern of change resulting from ostα deficiency is similar in both sexes but the magnitude of the changes for some parameters vary.

The role of ilbp in bile acid homeostasis is less understood. A previous study done on FXR-deficient mice [14], which lack ilbp secondary to FXR deficiency, suggested that ilbp was not needed for the recycling of bile acids in the EHC. In fact, kinetics studies using stable isotope labeled cholic acid as a tracer showed that cholic acid was efficiently reabsorbed in FXR-deficient mice causing the cholic acid pool to expand. As well, fecal excretion of bile acids and hepatic synthesis of bile acids were both increased in FXR-deficient mice. However, the precise role of ilbp in bile acid transport and metabolism could not be defined in this model because of FXR deficiency, which is needed for the proper regulation of many hepatic and intestinal genes involved in bile acid homeostasis.

In the present study, we found that targeted disruption of the gene for ilbp affected bile acid metabolism differently in male and female mice. In both sexes, the loss of ilbp did not significantly affect the bile acid pool size in the EHC. However, female mice exhibited enhanced fecal excretion of bile acids and increased cyp7a1 enzyme activity. Unexpectedly, cyp7a1 mRNA abundance was reduced in both sexes of *Fabp6^−/−^* mice. The reduction of cyp7a1 mRNA evident in both sexes of *Fabp6^−/−^* mice is likely not attributable to the action of FGF15 on *Cyp7a1* gene transcription in this study. The increase in cyp7a1 enzyme activity exhibited by female *Fabp6^−/−^* mice, or even by male *Fabp6^−/−^* mice which did not show a change in cyp7a1 enzyme activity despite the decrease in cyp7a1 mRNA, is likely mediated by posttranscriptional mechanism. Further studies are needed to clarify the exact cause of the observed disparity.

Tracing the fate of orally administered [^3^H]TCA permitted the visualization of bile acid transit originating from the gut. Interestingly, the loss of ilbp caused sex dimorphic effects on the retention and excretion of the exogenously administered radiolabeled bile acid. The recovery of [^3^H]TCA from blood, liver and gallbladder of both sexes of *Fabp6^−/−^* mice showed that ilbp is not required per se for the absorption and transport of bile acids. However, the increased amount of radiolabeled bile acid localized in the small intestine of male *Fabp6^−/−^* mice indicated that the loss of ilbp impairs the intestinal transit of bile acids in male mice. Yet, the changes in asbt protein abundance and ostα/ostβ abundance as predicted by ostβ mRNA in male and female *Fabp6^−/−^* mice were comparable. Contrary to male mice, the accumulation of bile acids in the small intestine as well as distal gut sac tissue of female *Fabp6^−/−^* mice was not observed. These results suggest that there may be another sex dimorphic mechanism for elimination of excess bile acids from enterocytes. This possibility was not explored in this study.

While ilbp by itself is not an absolute prerequisite for the transfer of bile acids from the intestine to the liver, it is required for efficient transport of bile acids by enterocytes in vivo. The residual intracellular bile acid transport activity in ilbp-deficient mice may be due to L-FABP activity. L-FABP has been shown to bind bile acids [10], [15]. However, L-FABP is normally distributed towards the proximal region of the small intestine where both asbt and ostα/ostβ are not well represented. Moreover, the abundance of L-FABP protein in the small intestine was not significantly altered by ilbp deficiency. Thus, based on our findings, it is evident that L-FABP is not fully capable of compensating for the loss of ilbp in vivo. It should be noted that the small intestine of female wild-type mice displays a higher concentration of ilbp than that of male wild-type mice. This may be needed for efficient transcellular transport in order to counteract the luminal release of bile acids in female mice.

In summary, the results obtained in this study provide evidence demonstrating that ilbp is needed for efficient movement of bile acids from the intestinal lumen to portal circulation by facilitating transcellular transport of bile acids in ileal enterocytes, and thereby establish a physiological role for ilbp in bile acid transport and homeostasis. Moreover, the sex-dimorphic modification of bile acid homeostasis resulting from the loss of ilbp suggests the possible existence of an alternative mechanism in female mice that facilitates the efficient excretion of excess bile acids from ileal enterocytes.

## Materials and Methods

### Creation of Ilbp-deficient Mice

BAC clones containing the murine *Fabp6* gene were isolated from a 129/Sv mouse embryonic stem genomic library and characterized by restriction enzyme mapping. A fragment containing all four exons of the *Fabp6* gene was subcloned into the pZero plasmid (Invitrogen, Burlington, ON). The pKO scrambler vector V915 (Lexicon Genetics Inc., Woodland, TX) was used to assemble the *Fabp6* targeting vector. Fragments containing exon I and exon IV of the *Fabp6* gene were subcloned into pV915 as depicted in Fig. 1. Gene cassettes encoding the neomycin phosphotransferase and HSV thymidine kinase genes (Lexicon Genetics Inc., Woodland, TX) were then cloned into the *Asc* I and *Rsr* II restriction sites, respectively, of the V915 vector. The targeting vector was electroporated into murine 129R1 embryonic stem cells. After selection, the cells were screened for the desired modification at the *Fabp6* gene by PCR using the following primers: forward primer: 5′-GCAGAGGATCAGGAGATTCAG-3′; reverse primer: 5′-GCGCATGCTCCAGACTGCCTTG-3′, which amplifies a fragment from the new junction created by homologous recombination at the 5′ end of the *Fabp6* gene. Embryonic stem cells that contained the modified *Fabp6* allele were injected into host mouse blastocysts and the resulting chimeric mice were mated to C57BL/6J mice (Jackson Laboratory, Bar Harbor, ME). The progeny were screened for the modified *Fabp6* allele by DNA blot analysis of tail biopsies. Mice (10–25 weeks old) used to characterize the phenotype of ilbp-deficiency were from a strain that had been backcrossed to the C57BL/6J background for at least 6 generations. Age matched C57BL/6J mice were used as wild-type controls. Mice were housed in a climate controlled environment and given free access to standard chow diet and water, unless stated otherwise. The use of animals in this study was approved by the animal care committees at the University of Alberta and McGill University.

### Analysis of Plasma

Plasma total cholesterol and triacylglycerol concentrations were measured using commercial diagnostic kits (Infinity Cholesterol and Infinity Triglycerides, respectively, Thermo Electron, Louisville, CO). Blood glucose concentration was measured using a Precision Xtra™ glucose monitor (MediSense, Bedford, MA). Commercial clinical assay kits were used to determine total bile acid concentration (Diagnostic Chemicals Limited, Charlottetown, PE), and ALT and AST activities (Stanbio Laboratory, Boerne, TX).

### Histological Analysis

Fresh tissues for histological evaluation were fixed in formalin, embedded in paraffin, cut into 6 µ thin sections and then stained with hematoxylin and eosin following standard procedures. Prepared slides were coded prior to assessment by the pathologist.

### RNA and Protein Analyses

Total RNA from liver and small intestine was isolated according to a previously described method [16]. RNA was assessed by gel electrophoresis to ensure integrity prior to analyses. For RNA blots, equal amounts of total RNA were separated by agarose gel electrophoresis, transferred to nylon membranes and then probed with [^32^P]-radiolabeled DNA probes. Membranes were washed at high stringency prior to detection of hybridized bands by autoradiography or by phosphorimaging. Quantitation of signals was done using the Quantity One analysis software (BioRad Laboratories Ltd, Mississauga, ON). For quantitative RT-PCR (qPCR), total cDNA was synthesized using MMLV reverse transcriptase (Agilent Technologies Canada Inc, Mississauga, ON), with both oligo(dT) and random hexamers as primers. Samples from each group were analyzed in duplicate using the BioRad CFX96 detection system with SYBR Green Supermix. Internal reference standards were GAPDH and villin mRNAs for intestinal RNA, and cyclophilin and β-actin mRNAs for liver RNA. The relative abundance of target mRNAs was calculated following the ΔΔCt method [17] using the Biorad CFX Manager software. The sequences of the primers used are listed in Table S1. Primer pairs were pre-tested for efficiency and specificity of amplification.

The presence of ilbp in homogenates of small intestine was determined by immunoblotting using a rabbit antiserum generated against recombinant murine ilbp [11]. Asbt was detected using a rabbit antiserum generated against a peptide based on the carboxyl terminal end of hamster asbt [18]. Proteins were separated by electrophoresis, transferred to membranes and then probed with rabbit antiserum to the target proteins. After washing, the membranes were reprobed with horseradish peroxidase conjugated anti-rabbit antibodies, washed, and then the immune complexes were visualized using the ECL Western Blotting System (GE Healthcare Life Sciences, Baie D'Urfé, QC). Quantitation was done by densitometry of bands in scanned films using Un-Scan-It 5.1 software (Silk Scientific Inc, Orem, UT).

### Enzyme activity assays and lipid analyses

Microsomes were prepared from liver homogenates and then assayed for cyp7a1 activity by following the conversion of [^14^C]cholesterol to [^14^C]7α-hydroxycholesterol as described previously [19]. The HMGR activity was assayed by measuring the conversion of [^14^C]HMG-CoA to [^14^C]mevalonate [20]. Hepatic unesterified cholesterol and cholesteryl esters concentrations were determined by gas chromatography [21].

### Bile Acid Pool Size, Speciation, Excretion and Absorption

To measure bile acid pool size, the total bile acids were extracted from liver, small intestine and gallbladder following a previously described method [22]. Gallbladder bile acids were analyzed by HPLC (Agilent 1200 Series) fitted with an evaporative light scattering detector (Agilent 1200 Series ELSD) [23]. Bile acid reference standards were obtained from Steraloids Inc (Wilton, NH).

To measure fecal bile acid excretion rate, mice were acclimated to individual housing in wire bottom cages and given free access to food and water. Stools were collected over 72 h, dried, weighed and then ground into a fine powder. The bile acids were extracted as described above and the amount of bile acids in the samples was determined using an enzymatic assay for total bile acids.

Bile absorption in vivo was determined by following the fate of a radiolabeled bile acid over a 24 h period in individually housed mice. [^3^H]TCA (10 µCi, 25 µmol in 0.1 ml) was administered by oral gavage. Treated mice were returned to clean cages and given free access to water. At 24 h, the mice were euthanized and the blood, liver, gallbladder, small intestine, large intestine, urine and stools were collected for analysis. The intestines were flushed and the luminal contents were combined with the stool samples. The tissues and dried stools were thoroughly homogenized in 50% (v/v) t-butanol [24]. The amount of radioactivity in aliquots diluted in 50% t-butanol of plasma, gallbladder bile and urine, and in clarified tissue and stool homogenates was determined by liquid scintillation spectrometry.

Comparisons between groups were done using commercial statistical software packages (SigmaStat 3.0 and GraphPad Prism 5) using parametric or nonparametric tests where appropriate. Data shown are mean ± SD unless indicated otherwise, and are representative of at least two independent experiments with 3–10 replicates. Differences were considered significant when *P*<0.05.

### Transport of Bile Acids in Everted Gut Sacs

The small intestine (∼30 cm) was removed, flushed with cold oxygenated Krebs Ringer Buffer (KRB), then gently everted using a stainless steel wire. The proximal third and the distal third were collected and then weighed. One end of each segment was tied off with a suture, and the sacs were filled with oxygenated KRB. The open ends were closed off with a suture and then the filled sacs were reweighed. Sacs were incubated for 30 min in oxygenated KRB prewarmed to 37°C, containing 25 uM [^3^H]TCA (specific activity = 70 mCi/mmol) (Perkin Elmer Inc., Woodbridge, ON) and 176,000 dpm/ml inulin [^14^C]carboxylic acid (specific activity = 2.47 mCi/mmol) (Perkin Elmer Inc., Woodbridge, ON). The assay was terminated by removing the sacs from the incubation buffer, then sacs were blotted dry and weighed. The serosal fluid was recovered and the tissues were blotted dry then solubilized in 1 N NaOH. The total amount of taurocholic acid associated with the serosal fluid and corresponding tissue was calculated based on the [^3^H] taurocholate and inulin [^14^C]carboxylic acid radioactivity as measured by liquid scintillation spectrometry, and on the final volume of fluid in the gut sacs and the mass of the tissue samples. The amount of inulin [^14^C]carboxylic acid radioactivity were similar for serosal fluid samples from proximal and distal gut sacs which were both substantially lower compared to mucosal fluid samples.

## Supporting Information

Figure S1 **Lipoprotein lipid profile of wild-type and ilbp-deficient mice.** Plasma samples from mice (n = 5 per group) were pooled and the lipoprotein lipid profile was analyzed as described in the Materials and Methods section. (A) Total cholesterol profile. (B) Total triacylglycerol profile.
(TIF)

Figure S2 **Morphology of distal small intestine of wild-type and ilbp-deficient mice.** Fresh tissue samples for histological evaluation were fixed in formalin, embedded in paraffin, cut into 6 µ thin sections and then stained with hematoxylin and eosin following standard procedures. Prepared slides were coded prior to assessment by the pathologist. Original magnification: 20×.
(TIF)

Figure S3 **Morphology of liver of wild-type and ilbp-deficient mice.** Fresh tissue samples for histological evaluation were fixed in formalin, embedded in paraffin, cut into 6 µ thin sections and then stained with hematoxylin and eosin following standard procedures. Prepared slides were coded prior to assessment by the pathologist. Original magnification: 20×.
(TIF)

Figure S4 **Tissue distribution of retained [3H]TCA in** ***Fabp6*** **^+/+^ and** ***Fabp6*** **^−/−^ mice (males, n = 4–5; females, n = 5–6) expressed as percent of total radioactivity.**
(TIF)

Figure S5 **Amount of tissue associated bile acids in the small intestine of** ***Fabp6*** **^+/+^ and** ***Fabp6*** **^−/−^ mice (males, n = 4; females, n = 4).** The small intestine was removed and flushed with an excess volume of cold saline. The organ was gently pressed to remove excess liquid in the lumen and then blotted dry with a tissue paper. The length (A) and weight (B) measurements were recorded. The amount of bile acids associated with the proximal, medial and distal thirds of the organ (C, males; D, females) was determined using a colorimetric total bile acid assay of tissue homogenates prepared in 50% t-butanol.
(TIF)

Figure S6 **The distribution of mRNAs for asbt (A) and ostα (B) along the proximal-to-distal axis of the small intestine of** ***Fabp6*** **^+/+^ (closed symbols) and** ***Fabp6*** **^−/−^ (open symbols) mice (males, n = 4; females, n = 4) was determined by qPCR.**
(TIF)

Table S1 **Primer sequences.**
(DOC)
