# Supplementary figures and images for: The Ileal Lipid Binding Protein Is Required for Efficient Absorption and Transport of Bile Acids in the Distal Portion of the Murine Small Intestine
Source: PLoS One. 2012 Dec 10;7(12):e50810. doi: 10.1371/journal.pone.0050810 (PMC3519535; doi:10.1371/journal.pone.0050810)

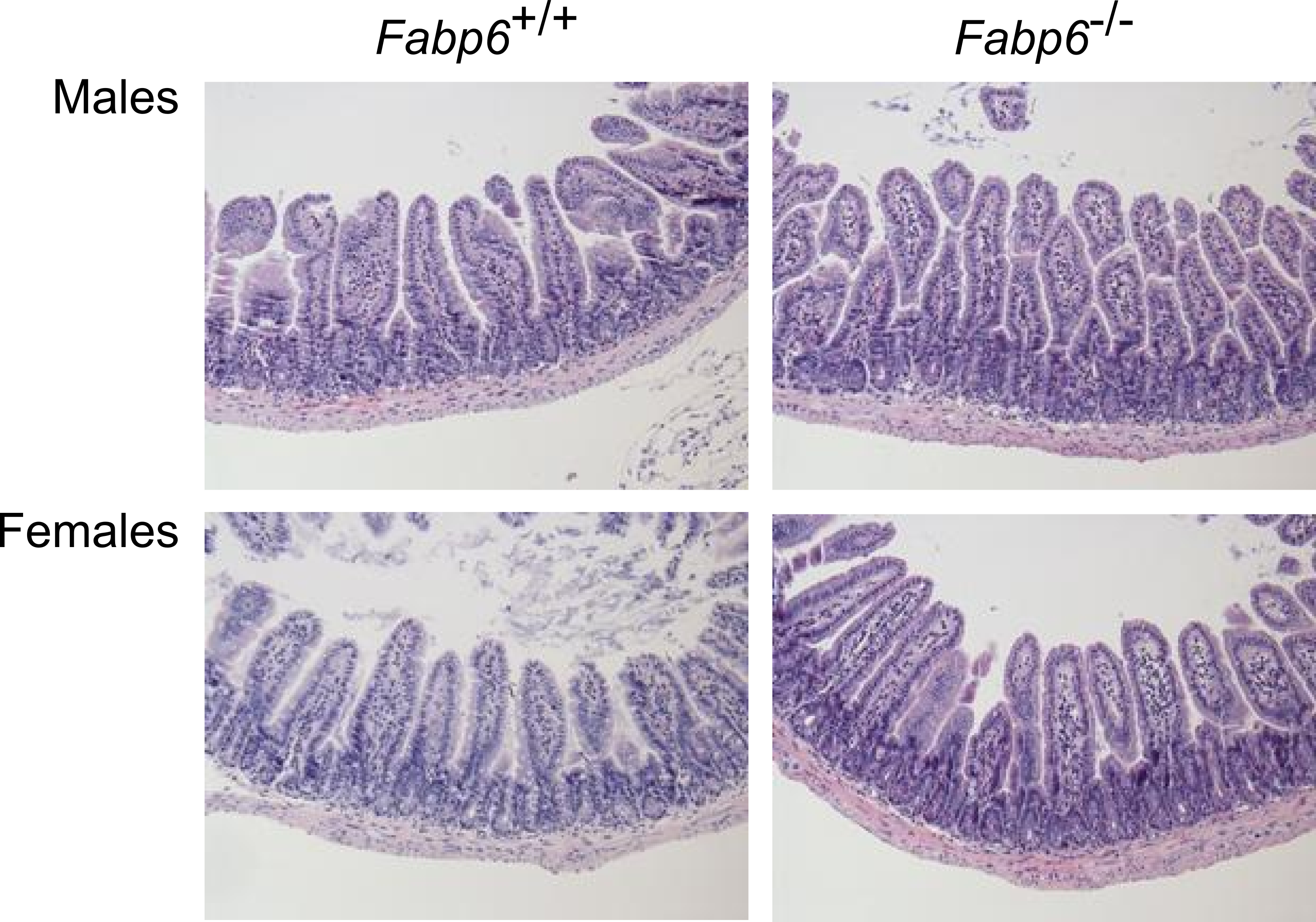

Supplement: Figure S2 — Morphology of distal small intestine of wild-type and ilbp-deficient mice. Fresh tissue samples for histological evaluation were fixed in formalin, embedded in paraffin, cut into 6 µ thin sections and then stained with hematoxylin and eosin following standard procedures. Prepared slides were coded prior to assessment by the pathologist. Original magnification: 20×. (TIF) [file pone.0050810.s002.tif]

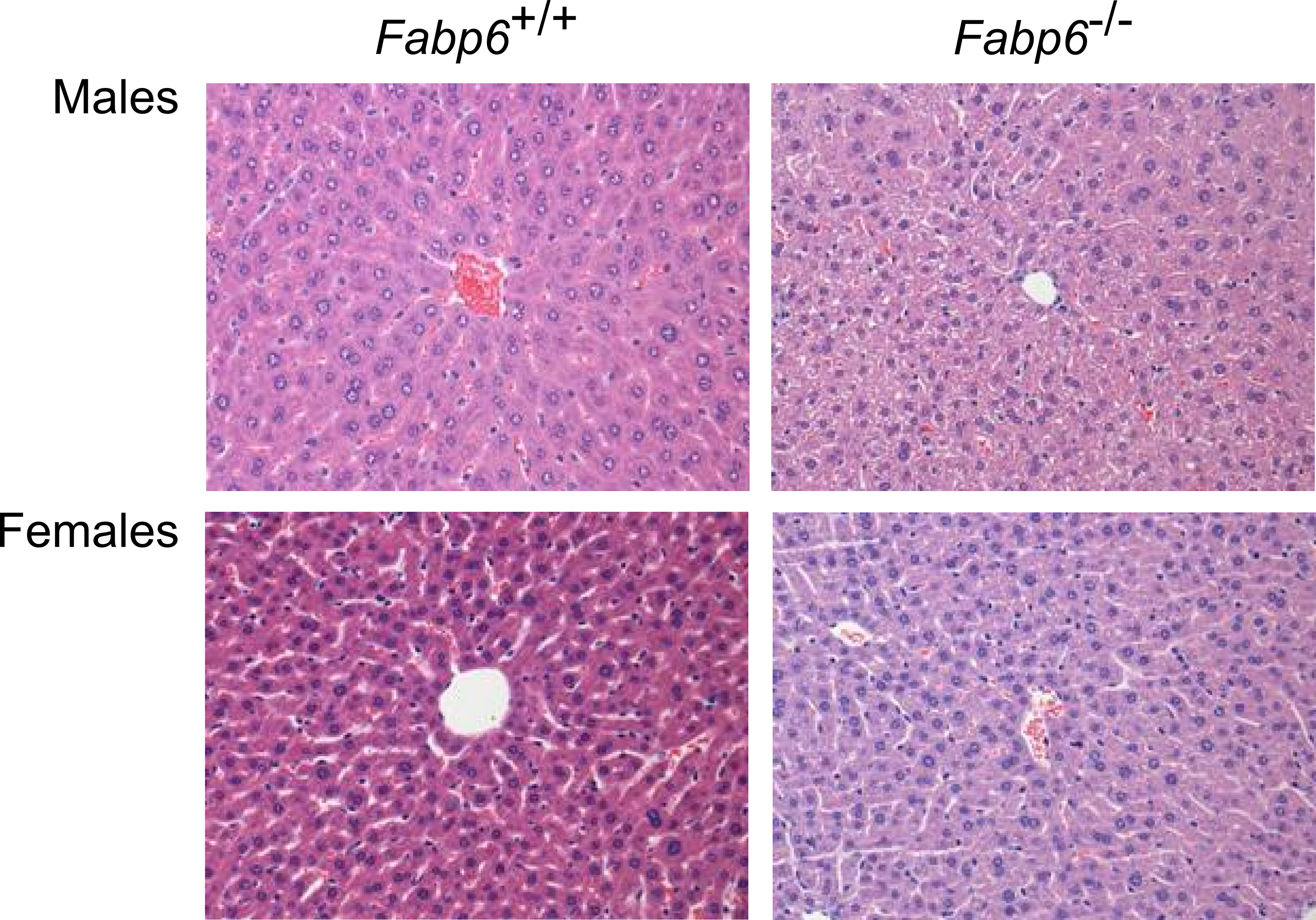

Supplement: Figure S3 — Morphology of liver of wild-type and ilbp-deficient mice. Fresh tissue samples for histological evaluation were fixed in formalin, embedded in paraffin, cut into 6 µ thin sections and then stained with hematoxylin and eosin following standard procedures. Prepared slides were coded prior to assessment by the pathologist. Original magnification: 20×. (TIF) [file pone.0050810.s003.tif]

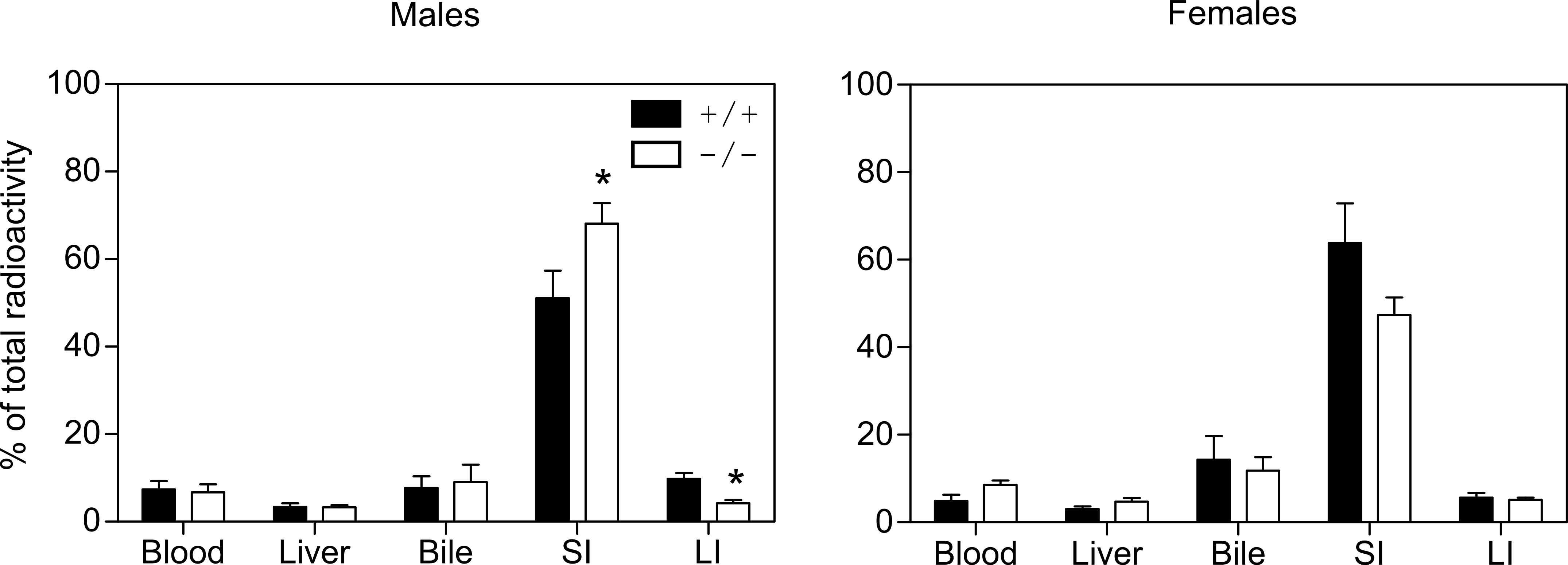

Supplement: Figure S4 — Tissue distribution of retained [3H]TCA in Fabp6 +/+ and Fabp6 −/− mice (males, n = 4–5; females, n = 5–6) expressed as percent of total radioactivity. (TIF) [file pone.0050810.s004.tif]

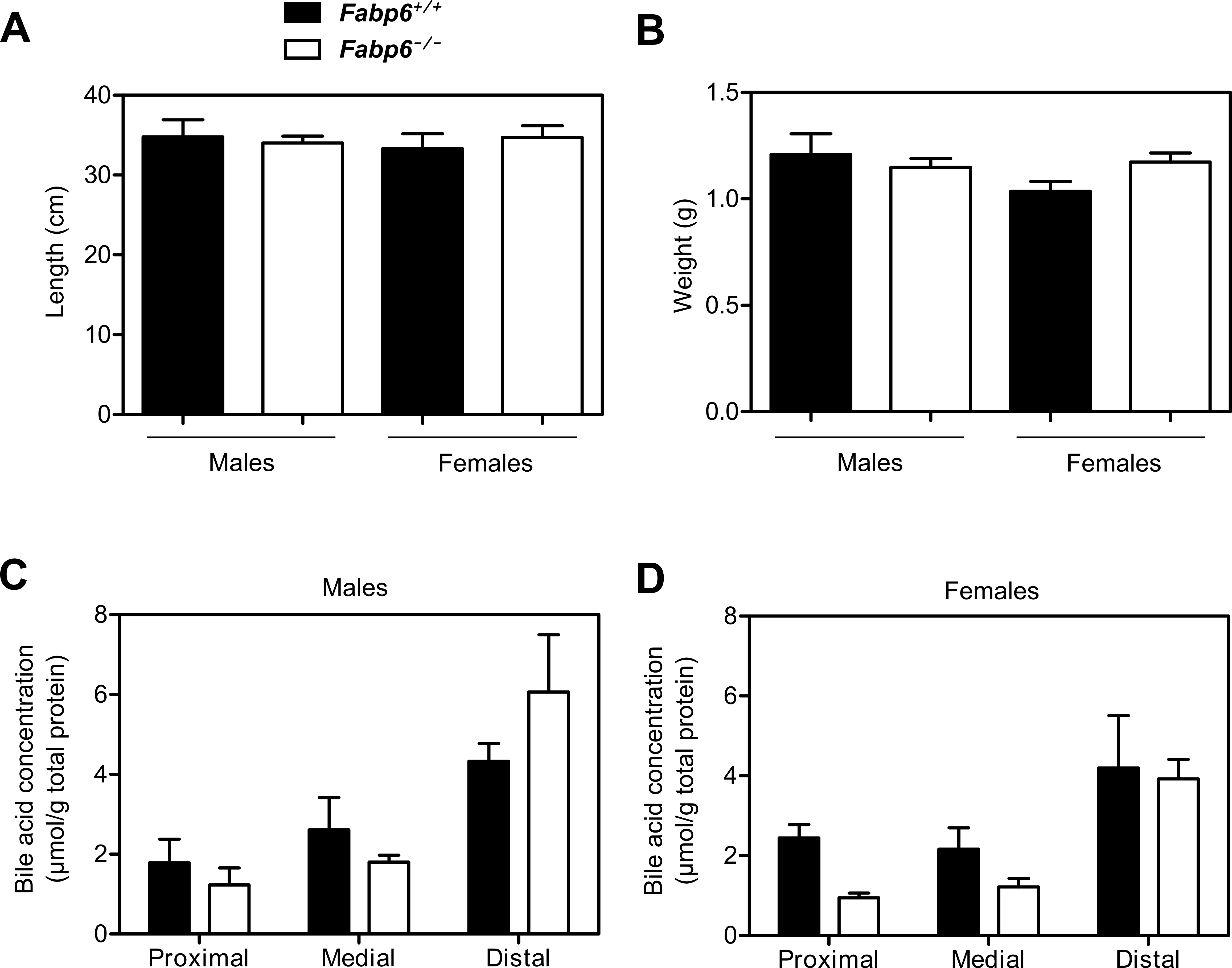

Supplement: Figure S5 — Amount of tissue associated bile acids in the small intestine of Fabp6 +/+ and Fabp6 −/− mice (males, n = 4; females, n = 4). The small intestine was removed and flushed with an excess volume of cold saline. The organ was gently pressed to remove excess liquid in the lumen and then blotted dry with a tissue paper. The length (A) and weight (B) measurements were recorded. The amount of bile acids associated with the proximal, medial and distal thirds of the organ (C, males; D, females) was determined using a colorimetric total bile acid assay of tissue homogenates prepared in 50% t-butanol. (TIF) [file pone.0050810.s005.tif]

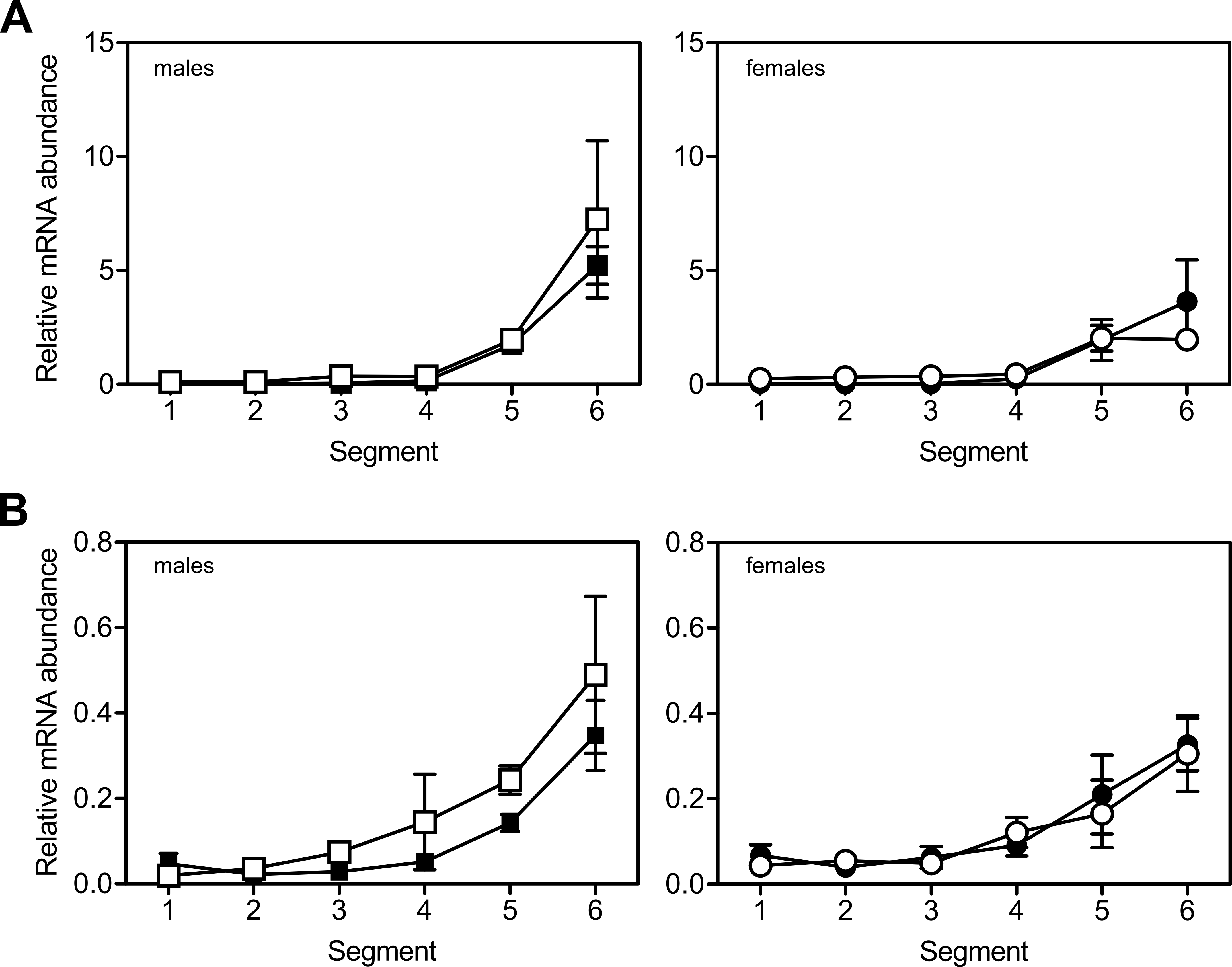

Supplement: Figure S6 — The distribution of mRNAs for asbt (A) and ostα (B) along the proximal-to-distal axis of the small intestine of Fabp6 +/+ (closed symbols) and Fabp6 −/− (open symbols) mice (males, n = 4; females, n = 4) was determined by qPCR. (TIF) [file pone.0050810.s006.tif]
