# Supplementary material for: The Ileal Lipid Binding Protein Is Required for Efficient Absorption and Transport of Bile Acids in the Distal Portion of the Murine Small Intestine
Source: PLoS One. 2012 Dec 10;7(12):e50810. doi: 10.1371/journal.pone.0050810 (PMC3519535; doi:10.1371/journal.pone.0050810)
Supplement: Table S1 — Primer sequences. (DOC) [file pone.0050810.s007.doc]

Table S1. Primer sequences

| Target | DNA sequence (5’→3’) |
| --- | --- |
| Abcb11 | F: CTGCCAAGGATGCTAATGCA  R: CGATGGCTACCCTTTGCTTCT |
| Asbt (Slc10a2) | F: TGGGTTTCTTCCTGGCTAGACT  R: TGTTCTGCATTCCAGTTTCCAA |
| β-actin | F: GAGACCTTCAACACCCC  GTGGTGGTGAAGCTGTAGCC |
| Cyp27a1 | F: GCCTTGCACAAGGAAGTGACT  R: CGCAGGGTCTCCTTAATCACA |
| Cyclophilin | F: TCCAAAGACAGCAGAAAACTTCG  R: TCTTCTTGCTGGTCTTGCCATTCC |
| Cyp7a1 | F: AGCGCTGTCTGGGTCACGGAAGG  R: GTAGTAGTTCCTCCGAGACGCCG |
| Cyp8b1 | F: GTCACTCCATGGCTTTCCGG  R: CTTTAGGCCCTAGCATCACC |
| FGF15 | F: ATGGCGAGAAAGTGGAACGG  R: GGACCAGCGGAGTACAGGT |
| FXR (Nr1h4) | F: CTTGATGTGCTACAAAAGCTGTG  R: ACTCTCCAAGACATCAGCATCTC |
| Gapdh | F: GAACGCAAAGCTGAAGTGAGACT  R: TCATTACGCTTGCACTGTTGGT |
| Hmgr (Hmgcr) | F: AGCTTGCCCGAATTGTATGTG  R: TCTGTTGTGAACCATGTGACTTC |
| L-FABP (Fabp1) | F: Tgcagagccaggagaactttgagcca  R: ccccagggtgaactcattgcggac |
| Ostα | F: GTCTCAAGTGATGAACTGCCA  R: TTGAGTGCTGAGTCCAGGTC |
| Ostβ | F: GTATTTTCGTGCAGAAGATGCG  R: TTTCTGTTTGCCAGGATGCTC |
| Ntcp/Slc10a1 | F: ATGACCACCTGCTCCAGCTT  R: GCCTTTGTAGGGCACCTTGT |
| Srebp-1c (Srebf1) | F: GGAGCCATGGATTGCACATT  R: GCTTCCAGAGAGGAGGCCAG |
| Srebp-2 (Srebf2) | F: GCAGCAACG GGACCATTCT  R: CCCCAT GACTAAGTCCTTCAACT |
| Villin | F: TCAAAGGCTCTCTCAACATCAC  R: AGCAGTCACCATCGAAGAAGC |
